# Supplementary material for: OligoSeq: Rapid nanopore-sequencing of single-stranded oligonucleotides
Source: PLoS One. 2026 Sep 3;21(9):e0355056. doi: 10.1371/journal.pone.0355056 (PMC13541252; doi:10.1371/journal.pone.0355056)
Supplement: Supplementary file 1 [file pone.0355056.s001.pdf]

SI file: Sequencing costs per platform and strand-aware alignment using anchor-based read filtering

S1 Sequencing costs per platform

S1.1 ONT sequencing costs

For example, running a sample through using ONT requires the purchase of flow cells or flongles (in this study, R10 flongles from ONT were used). The cost of each flongle is US$67.5 while the native barcoding kit (SQK-NBD114.24 Native Barcoding Kit 24 V14) used in this work costs about US$699.00 for a set of 6 reactions with each reaction able to barcode up to 24 different sequences, which falls to US$4.85 per barcode. Thus the total cost of sequencing one single oligo falls to US$7.7 per oligo (provided a full set of 24 oligoes are sequenced per flongle). To this is important to add the cost of reagents to convert the single-stranded oligonucleotides to double-stranded which costs US$1.28 per reaction (£0.95 according to current NEB UK prices for the reagents involved making a maximum of US$9 per oligo sequenced, calculated as follows:

- ONT R10 Flongle: US$67.50 or £51.00 (per flow cell), amortized to US$2.81 or £2.13 per sample when 24 samples are multiplexed
- ONT Native Barcoding Kit (SQK-NBD114.24): US$699.00 or £529.00 for 6 reactions (24 barcodes), equating to US$4.85 or £3.67 per sample
- Double-stranding reagents (NEB): US$1.28 or £0.95 per reaction

Total cost per oligo: US$8.94 or £6.75, when sequencing 24 samples per Flongle, considering an exchange rate of 1.33 US$/£ (as of December 2025).

S1.2 PacBio sequencing costs

The SMRTbell® adapter index plate 96A costs £571 for 96 samples, equating to £5.95 per sample. The SMRTbell® Prep Kit 3.0 (PacBio® 102-182-700), used to circularize DNA, costs £1,598 and supports 24 libraries of 96 samples each, enabling up to 2,304 samples with a per-sample cost of £0.69 if fully utilized. The Binding Kit 3.1 and Cleanup Beads 3.1 (PacBio® 102-194-200), required for polymerase binding, is priced at £1,434, also supporting 2,304 samples and reducing the per-sample cost to £0.62 under full usage. Additional sequencing costs include a flow cell at £3,584 for four runs (£896 per run), translating to £9.33 per sample; SMRTcell Oil 4x (5 tubes for 4 runs) costs £158, adding £0.411 per sample; lids for oil tubes cost £33 for 20 runs, adding £0.086 per sample; and the Sequel II Sequencing Kit 2.0 for four flow cells costs £761, adding £1.98 per run. Additional overheads include costs for specific plates, tips, and lids required for PacBio equipment operation. Summing all up, the total cost per sample, excluding final overheads (plates, tips, lids), is £18.45, calculated as follows:

- SMRTbell® adapter index plate 96A: £5.95 or US$7.91
- SMRTbell® Prep Kit 3.0: £0.69 or US$0.92
- Binding and Cleanup Kit 3.1: £0.62 or US$0.82
- Flow cell: £9.33 or US$12.41
- SMRTcell Oil: £0.411 or US$0.55

- Oil lid: £0.086 or US$0.11
- Sequel II Sequencing Kit 2.0: £1.98 or US$2.63

Which, based on a £-to-USD exchange rate of 1.33 (December 2025), costs an average (much) greater than £18.45 or US$24.55 per sample to use PacBio (in the unlikely event that over 2,000 samples are ready to be processed).

S1.3 Illumina sequencing costs

This workflow for Illumina sequencing is similar to the one described for ONT, which consists in (1) adapter ligation and (2) barcoding ligation, and also needs a proprietary flow cell for use. This workflow also needs double-stranding of single-stranded oligonucleotides which includes the overhead of US$1.28 per reaction/sample (£0.95 according to current NEB UK prices for the reagents involved, For sample specific-preparation for IllUmina sequencing, the NEBNext UltraExpress® DNA Library Prep Kit is used, that includes dA tailing and end-repair, adapter ligation, PCR enrichment and cleanup, the cost for a kit capable of processing 96 samples is £1,880.00 which falls down to £19.58 per sample. For multiplexing 96 reactions for a separate sample per barcode, it is possible to use (similarly as with the ONT native barcoding kit) the barcoding kit corresponding to Illumina (NEBNext® Multiplex Oligos for Illumina® (96 Unique Dual Index Primer Pairs)) where a whole plate is £570.00 which would be £5.93 per sample. Once the sample is prepared using these reagents, a flow cell is needed for sequencing which costs no less than USD129.00 and using an external serivce provider Novogene (Cambridge, United Kingdom) costed £111.36.

- i1 Flow Cell: US$129.00 or £96.99 (per flow cell), amortized to US$1.78 or £1.34 per sample when 96 samples are multiplexed
- NEBNext UltraExpress® DNA Library Prep Kit: US$2,500.40 or £1,880.00 for 96 reactions, equating to US$26.04 or £19.58 per sample
- NEBNext® Multiplex Oligos for Illumina® (96 Unique Dual Index Primer Pairs): US$758.10 or £570.00 for 96 reactions, equating to US$7.89 or £5.93 per sample
- Double-stranding reagents (NEB): US$1.28 or £0.95 per reaction

Which, based on a £-to-US$ exchange rate of 1.33 (December 2025), costs an average (much) greater than £ $27.80 or US$36.99 per sample to use Illumina iSeq.

S1.4 HPLC/MS sequencing costs

Conversely, using an Agilent Suite HPLC/MS service at Imperial College London which runs oligonucleotides on the Oligo LC/MS - 1290 Infinity III with Pro iQ Plus SQ MS (Agilent Technologies, California, USA), costs no less than US$43 per hour and at least (with 0.5 hours dedicated to initial equipment start-up, heat up and sample preparation and 0.33 hours per oligo processed) will be required to run 3 different oligo samples. Without considering the reagents needed for operation and maintenance. Thus the cost of running one oligo falls to at least US$21.5 + US$14.33 = US$36 per oligo. Still, without considering equipment start-up timings, the cost of adding up one more oligo in the HPLC/MS analysis is US$14.33, which is a 100 % greater than using ONT technology, and the HPLC/MS in question is only able to resolve molecules by molecular mass which adds further overhead complexity to resolve the actual DNA sequences, increasing its cost therefrom. Thus the only foreseeable advantage could be the lack of sample preparation—as the ssDNA oligo samples can be run directly on the

HPLC/MS equipment—which might help save time, however the need to run the mobile phases and heat the colum for HPLC/MS overrides this seeming advantage of a lack of sample-prep, thus the summary costs follow below as:

- HPLC/MS instrument time (start-up): US$21.50 or £16.10 (0.5 hours at US$43/hour)
- HPLC/MS run per oligo: US$14.33 or £10.73 (0.33 hours per oligo at US$43/hour)

Total cost per oligo: US$35.83 or £26.83, excluding reagents and maintenance considering an exchange rate: 1.33 US$/£.

S2 Strand-aware alignment using anchor-based read filtering

Script S1. Code for the strand-aware alignment using anchor-based read filtering for the assessment of oligo quality.

```
"""
###␣This␣code␣does:
#####␣Match␣forward␣reads␣to␣the␣left␣anchor,
#####␣Match␣reverse␣reads␣(revcomp)␣to␣the␣right␣anchor,
#####␣Combine␣them␣into␣one␣sequence␣logo␣plot␣to␣show␣varaibility
#####␣Usage␣example:
␣␣␣␣␣␣␣␣python␣strand_aware_logo.py␣--fastq_dir␣fastq_pass␣--reference␣reference.fa␣--anchor_len␣20␣--output␣logo.png
###␣author:␣Robert␣Ramirez-Garcia
"""
import os
import argparse
from collections import Counter
from Bio import SeqIO
from Bio.Seq import Seq
import pandas as pd
import matplotlib.pyplot as plt
import logomaker
import matplotlib.patches as patches

def read_fasta(fasta_file):
    ### Reads a .fasta file and returns the sequence
    with open(fasta_file, 'r') as file:
        seq = []
        for line in file:
            if not line.startswith('>'):
                seq.append(line.strip())
    return ''.join(seq)

def get_sequences_from_fastq_folder(folder):
    ### Reads all sequences from all .fastq files within a folder
    seqs = []
    for fname in os.listdir(folder):
        if fname.endswith('.fastq') or fname.endswith('.fq'):
            for record in SeqIO.parse(os.path.join(folder, fname), "fastq"):
                seqs.append(str(record.seq))
    return seqs

def reverse_complement(seq):
    ### Return the reverse complement of a DNA sequence
    return str(Seq(seq).reverse_complement())

def anchor_extract(reference, sequence, anchor_len, mode):

    ### Extracts and aligns sequence to reference based on anchor mode.
    ### Returns a sequence of reference length, padded with '-' if needed.
    ### Modes: 'both', 'left', 'right'

    ref_len = len(reference)
    ref_left = reference[:anchor_len]
    ref_right = reference[-anchor_len:]

    if mode == 'both':
        if sequence.startswith(ref_left) and sequence.endswith(ref_right):
            mid_seq = sequence[anchor_len:len(sequence)-anchor_len]
            new_seq = ref_left + mid_seq + ref_right
            return new_seq[:ref_len].ljust(ref_len, '-')
    elif mode == 'left':
        if sequence.startswith(ref_left):
            mid_seq = sequence[anchor_len:]
            new_seq = ref_left + mid_seq
            return new_seq[:ref_len].ljust(ref_len, '-')
    elif mode == 'right':
        if sequence.endswith(ref_right):
            mid_seq = sequence[:len(sequence)-anchor_len]
            new_seq = mid_seq + ref_right
            return new_seq[-ref_len:].rjust(ref_len, '-')
    return None

def get_most_abundant_sequence(seq_list):
    if not seq_list:
        return ""
```

```
    return Counter(seq_list).most_common(1)[0][0]

def create_sequence_logo(seq_strings, reference, output_file="sequence_logo.png"):
    max_length = len(reference)
    alphabet = ['A', 'C', 'G', 'T', '-']
    counts_matrix = pd.DataFrame(0, index=range(max_length), columns=alphabet)
    for seq in seq_strings:
        for pos, char in enumerate(seq):
            if char in alphabet:
                counts_matrix.loc[pos, char] += 1
    freq_matrix = counts_matrix.div(counts_matrix.sum(axis=1), axis=0).fillna(0)
    consensus = ''
    for pos in range(max_length):
        max_freq = freq_matrix.iloc[pos].max()
        if max_freq > 0.5:
            consensus += freq_matrix.iloc[pos].idxmax()
        else:
            consensus += 'N'

    colour_code = {
        'A': '#008000', # Green
        'C': '#0000FF', # Blue
        'G': '#FFA500', # Orange
        'T': '#FF0000', # Red
        '-': '#808080', # Gray
        'N': '#808080'
    }

    most_abundant = get_most_abundant_sequence(seq_strings)

    fig, (ax_logo, ax_abundant, ax_ref, ax_cons) = plt.subplots(
        4, 1, figsize=(15, 10),
        gridspec_kw={'height_ratios': [4, 1, 1, 1]},
        sharex=True
    )

    logo = logomaker.Logo(freq_matrix, ax=ax_logo, color_scheme=colour_code)
    logo.style_spines(visible=False)
    ax_logo.set_ylabel('Frequency')
    ax_logo.set_title('Sequence␣Logo')

    for pos, char in enumerate(most_abundant):
        if char in colour_code:
            colour = colour_code[char]
            rect = patches.Rectangle((pos-0.5, 0), 1, 1, facecolor=colour, alpha=0.6)
            ax_abundant.add_patch(rect)
            ax_abundant.text(pos, 0.5, char, ha='center', va='center', fontsize=10, fontweight='bold', color='white')
    ax_abundant.set_ylim(0, 1)
    ax_abundant.set_yticks([])
    ax_abundant.set_title('Most␣Abundant␣Sequence')
    ax_abundant.set_frame_on(False)

    for pos, char in enumerate(reference.ljust(max_length, '-')):
        if char in colour_code:
            colour = colour_code[char]
            rect = patches.Rectangle((pos-0.5, 0), 1, 1, facecolor=colour, alpha=0.6)
            ax_ref.add_patch(rect)
            ax_ref.text(pos, 0.5, char, ha='center', va='center', fontsize=10, fontweight='bold', color='white')
    ax_ref.set_ylim(0, 1)
    ax_ref.set_yticks([])
    ax_ref.set_title('Reference␣Sequence')
    ax_ref.set_frame_on(False)

    for pos, char in enumerate(consensus):
        if char in colour_code:
            colour = colour_code[char]
            rect = patches.Rectangle((pos-0.5, 0), 1, 1, facecolor=colour, alpha=0.6)
            ax_cons.add_patch(rect)
            ax_cons.text(pos, 0.5, char, ha='center', va='center', fontsize=10, fontweight='bold', color='white')
    ax_cons.set_ylim(0, 1)
    ax_cons.set_yticks([])
    ax_cons.set_xlabel('Position')
    ax_cons.set_title('Consensus␣Sequence')
    ax_cons.set_frame_on(False)

    plt.tight_layout()
    plt.savefig(output_file, dpi=300)
    print(f"Most␣abundant␣sequence:␣{most_abundant}")
    print(f"Consensus␣sequence:␣{consensus}")
    print(f"Sequence␣logo␣saved␣as␣{output_file}")

if __name__ == "__main__":
    parser = argparse.ArgumentParser(description="Strand-aware␣anchor␣alignment␣and␣sequence␣logo␣generator.")
    parser.add_argument("--fastq_dir", type=str, default="fastq_pass", help="Directory␣with␣.fastq␣files")
    parser.add_argument("--reference", type=str, required=True, help=".fasta␣/␣.fa␣file␣with␣reference␣sequence")
    parser.add_argument("--anchor_len", type=int, default=20, help="Length␣of␣anchor␣region␣(nt)")
    parser.add_argument("--output", type=str, default="sequence_logo.png", help="Output␣image␣filename")
    args = parser.parse_args()

    reference = read_fasta(args.reference)
    all_seqs = get_sequences_from_fastq_folder(args.fastq_dir)
    print(f"Total␣sequences␣read:␣{len(all_seqs)}")

    selected = []

    for seq in all_seqs:
        aligned = anchor_extract(reference, seq, args.anchor_len, mode="left")
        if aligned:
            selected.append(aligned)
            continue

        rev_seq = reverse_complement(seq)
        aligned_rc = anchor_extract(reference, rev_seq, args.anchor_len, mode="right")
        if aligned_rc:
```

```
        selected.append(aligned_rc)

print(f"Sequences␣passing␣anchor␣filter:␣{len(selected)}")
if not selected:
    print("No␣sequences␣passed␣the␣anchor␣filter.")
    exit(1)

### Precomputes most abundant and consensus for clean reporting
most_abundant = get_most_abundant_sequence(selected)

max_length = len(reference)
counts_matrix = pd.DataFrame(0, index=range(max_length), columns=['A', 'C', 'G', 'T', '-'])
for seq in selected:
    for pos, char in enumerate(seq):
        if char in counts_matrix.columns:
            counts_matrix.loc[pos, char] += 1
freq_matrix = counts_matrix.div(counts_matrix.sum(axis=1), axis=0).fillna(0)

consensus = ''
for pos in range(max_length):
    max_freq = freq_matrix.iloc[pos].max()
    if max_freq > 0.5:
        consensus += freq_matrix.iloc[pos].idxmax()
    else:
        consensus += 'N'

### Outputs all expected logs
print(f"Most␣abundant␣sequence:␣{most_abundant}")
print(f"Reference␣sequence␣used␣in␣plot:␣{reference}")
print(f"Consensus␣sequence:␣{consensus}")

### Creates and saves the logo
create_sequence_logo(selected, reference, args.output)
```
